# Subjective Cognitive Fatigue and Autonomic Abnormalities in Multiple Sclerosis Patients

**DOI:** 10.3389/fneur.2017.00475

**Published:** 2017-09-13

**Authors:** Carina Sander, Helmut Hildebrandt, Hans-Peter Schlake, Paul Eling, Katrin Hanken

**Affiliations:** ^1^Institute of Psychology, University of Oldenburg, Oldenburg, Germany; ^2^Rehabilitation Center Wilhelmshaven, Wilhelmshaven, Germany; ^3^Klinikum Bremen-Ost, Department of Neurology, Bremen, Germany; ^4^Donders Institute for Brain, Cognition and Behaviour, Radboud University Nijmegen, Nijmegen, Netherlands

**Keywords:** multiple sclerosis, cognitive fatigue, Fatigue Scale for Motor and Cognitive Functions, autonomic failures, COMPASS-31, vagus nerve, inflammation

## Abstract

**Background:**

Cognitive fatigue and autonomic abnormalities are frequent symptoms in MS. Our model of MS-related fatigue assumes a shared neural network for cognitive fatigue and autonomic failures, i.e., aberrant vagus nerve activity induced by inflammatory processes. Therefore, they should occur in common.

**Objective:**

To explore the relationship between cognitive fatigue and autonomic symptoms in MS patients, using self-reported questionnaires.

**Methods:**

In 95 MS patients, cognitive fatigue was assessed with the Fatigue Scale for Motor and Cognitive Functions and autonomic abnormalities with the Composite Autonomic Symptom Scale-31 (COMPASS-31). We used exploratory correlational analyses and hierarchical regression analysis, controlling for age, depressive mood, disease status, and disease duration, to analyze the relation between autonomic abnormalities and cognitive fatigue.

**Results:**

The cognitive fatigue score strongly correlated with the COMPASS-31 score (*r* = 0.47, *p* < 0.001). Regression analysis revealed that a model, including the COMPASS-31 domains: pupillomotor, orthostatic intolerance, and bladder, best predict the level of cognitive fatigue (*R*^2^ = 0.47, *p* < 0.001) after forcing the covariates into the model.

**Conclusion:**

In MS patients, cognitive fatigue and autonomic dysfunction share a proportion of variance. This supports our model assuming that fatigue might be explained at least partially by inflammation-induced vagus nerve activity.

## Introduction

Fatigue and autonomic abnormalities, such as cardiovascular or bladder dysfunctions, are frequent symptoms in multiple sclerosis decreasing a patient’s quality of life. Both, fatigue and autonomic abnormalities, occur in early stages of MS and some studies reported an association between fatigue and autonomic abnormalities in MS patients which might indicate a common pathological mechanism underlying these symptoms (1–5).

Recently, Hanken et al. (6) developed a model for explaining fatigue in MS patients. According to this model, increased bodily inflammation plays a major role in the generation of MS-related fatigue. It assumes that proinflammatory cytokines activate the afferents of the vagus nerve which convey information about bodily inflammation to interoceptive brain areas, such as the nucleus tractus solitarius, the hypothalamus, the insular cortex, the anterior cingulate cortex, and the amygdala. According to the model, this inflammation-induced activity in interoceptive brain areas also is associated with the generation of the feeling of fatigue in MS patients. It should be noted that apart from this model, there are other models trying to explain the emergence of cognitive fatigue differently, but we will focus on this model in the following way: trying to find underpinning aspects.

The nervous structures that play an important role in the model of MS-related fatigue such as the vagus nerve and interoceptive brain areas also are considered to be the main components of the autonomic nervous system (7). Interoceptive brain areas integrate visceral sensory information and regulate autonomic functions, whereas efferents of the vagus nerve form a major component of the parasympathetic nervous system controlling several autonomic functions. Moreover, proinflammatory cytokines are considered to have an influence on the control of autonomic functioning (8, 9) as they activate the afferent vagus nerve that subsequently activates efferents of the vagus nerve. This might result in parasymapathetic overactivity causing a disruption of autonomic control. Those autonomic functions that are under direct control of the efferent vagus nerve should be mostly disrupted by this overactivity (9).

Hence, we assume that common pathological mechanisms, such as chronic peripheral inflammation and a resulting overactivity of the vagus nerve, generate fatigue as well as autonomic abnormalities in MS patients.

The aim of this study was to explore the relationship between subjective cognitive fatigue and the global amount of self-reported autonomic abnormalities in MS patients. Moreover, we wanted to investigate which autonomic abnormalities best predict the level of MS-related cognitive fatigue.

## Materials and Methods

**Figure 1:**
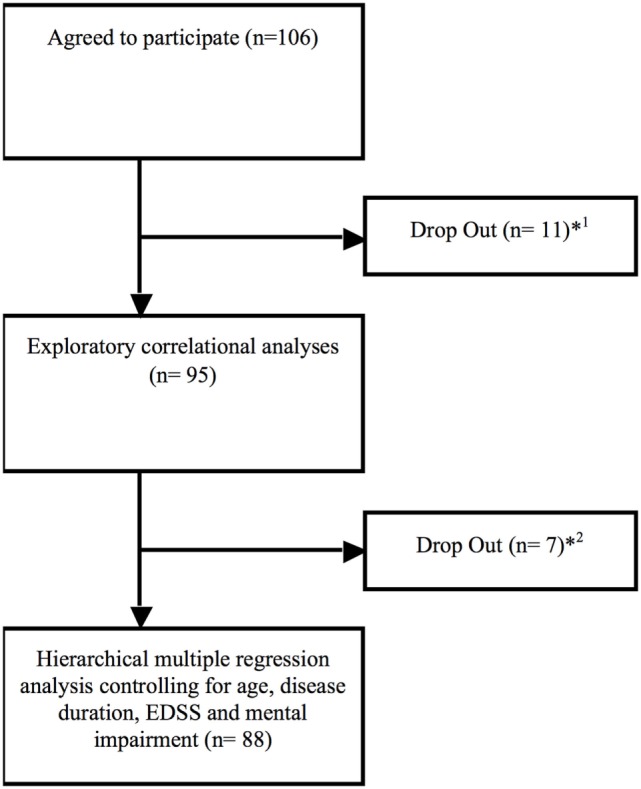
Drop out flow chart. ^*1^Eight participants did drop out immediately due to early departure (*n* = 8), missing data of the COMPASS (*n* = 2) and the Fatigue Scale for Motor and Cognitive Functions (*n* = 1) due to not being consent to answer single questions out of personal reasons.^*2^Drop out due to missing data about disease duration (*n* = 3; no clear date in personal medical history), Expanded Disability Status Scale (*n* = 2; not assessed by the medical staff) and Beck Depression Inventory Scale (*n* = 2; no consent to complete this questionnaire).

106 MS patients with a relapsing–remitting (rr MS; 57%), a secondary progressive (sp MS; 22%), or a primary progressive (pp MS; 21%) disease course according to the McDonald criteria (10) participated in this study lasting from May 2016 to June 2017. 11 participants did drop out in analyses due to early departure and three due to missing answers in the Composite Autonomic Symptom Scale-31 (COMPASS-31) or the Fatigue Scale for Motor and Cognitive Functions (FSMC). Additional seven participants did drop out due to missing data of disease duration, Expanded Disability Status Scale (EDSS), and Beck Depression Inventory Scale (BDI) (see Figure 1 and Table 1).

**Table 1:** Data of the complete drop outs.

	Drop outs
Number (*n*)	11
Age^a^	54.10 ± 11.46
Gender (male:female)	3:7
EDSS score^a^	4.6 ± 2.28
Disease course	pp MS: 3 rr MS: 5 sp MS: 2

*^a^Mean ± SD*.

*EDSS, Expanded Disability Status Scale; n, number; pp, primary progressive; rr, relapsing–remitting; sp, secondary progressive; MS, multiple sclerosis*.

Patients have been inpatients of the Rehabilitation Center Wilhelmshaven, Wilhelmshaven, Germany and were interviewed at the beginning of their rehabilitation program. Individuals with a MS relapse or using corticosteroids during the last 4 weeks, under legal care and/or with a diagnosis of any other neurodegenerative disease were excluded from the study. The study was approved by the ethical board of the Medical Society in Bremen and written informed consent was obtained from participants.

To assess the level of subjective cognitive fatigue, we used the cognitive fatigue subscore of the FSMC (11). The FSMC is a self-report questionnaire consisting of 20 questions of which 10 questions assess the severity of cognitive fatigue. The cognitive fatigue subscore ranges from 10 to 50 and a score higher or equal to 28 indicates moderate cognitive fatigue. To assess the severity of autonomic symptoms, we used the COMPASS-31, consisting of 31 questions (12). The questions assess the severity of autonomic symptoms in six different domains, including orthostatic intolerance, vasomotor, secretomotor, gastrointestinal, bladder, and pupillomotor activity. The total score ranges from 0 to 100, with higher scores reflecting more severe autonomic dysfunctions (cutoff value 32.5). In addition, the severity of depressive symptoms was assessed using the BDI (13). We used the items A-O, the mental items, to calculate the score for mood impairment, whereas the P-U items (sleep, tiredness, body weight, loss of sexual interest, and somatic concerns) reflect the somatic score.

Exploratory (Spearman correlation) and partial correlational analyses were performed to investigate the relation between cognitive fatigue, autonomic dysfunctions, and clinical data.

Hierarchical multiple regression analysis was used to analyze the prediction of cognitive fatigue status on the basis of autonomic dysfunctions. In a first step, the variables age, disease duration (time since first diagnosis), EDSS, and the mental BDI score were included in the regression analysis as control variables. In a second step, we added the autonomic domain scores (orthostatic intolerance, vasomotor, secretomotor, gastrointestinal, bladder, and pupillomotor domain score) to see if and which of them would improve the prediction. For this second block, forward regression analysis was used.

To check for the influence of autonomic disturbances on motor fatigue, the same regression analysis was performed with the motor fatigue subscore of the FSMC as dependent variable.

Furthermore, we checked for differences between the rr MS, sp MS, and pp MS patients in the cognitive score of the FSMC and the scales of the COMPASS-31 using one-way ANOVA and Kruskal–Wallis *H* test, respectively.

## Results

95 patients (male:female ratio = 30:65; mean age = 49; 57.6% rr MS; 22.8% sp MS; 19.6% pp MS) provided responses to all items of the FSMC and the COMPASS-31. These patients had a mean disease duration of 13 years and a mean EDSS score of four. Overall, 66% of patients presented a cognitive fatigue subscore equal or above 28 indicating moderate cognitive fatigue, 17% presented autonomic abnormalities (COMPASS-31 score equal or higher to 32.5) and 32% showed signs of depression (BDI equal or higher to 14; see Tables 2 and 3).

**Table 2:** Data of the study group.

	MS study group
Number (*n*)	95
Gender (male:female)	30:65
Age^a^	49.17 ± 10.1
Disease duration (years)^a^	12.7 ± 9.7
EDSS score^a^	3.8 ± 2.3
Cognitive fatigue subscore of the FSMC^a^	31.5 ± 10.0
BDI total score^a^	11.3 ± 6.4
COMPASS-31 score^a^	21.3 ± 11.5
Orthostatic intolerance subscore COMPASS-31^a^	4.5 ± 3.1
Vasomotor subscore COMPASS-31^a^	1.1 ± 1.6
Secretomotor subscore COMPASS-31^a^	1.5 ± 1.7
Gastrointestinal subscore COMPASS-31^a^	6.2 ± 5.1
Bladder subscore COMPASS-31^a^	2.7 ± 2.5
Pupillomotor subscore COMPASS-31^a^	5.3 ± 3.7
Disease modifying treatment	None: 54 Natalizumab: 4 Galtirameracetate: 3 Fingolimod: 3 Interferon-beta: 13 Teriflunomide: 8 Dimethylfumarate: 9 Mitoxantrone: 1
Spasmolytics (Fampyra or Baclofen)	20

*^a^Mean ± SD*.

*BDI, Beck Depression Inventory; COMPASS-31, Composite Autonomic Symptom Scale-31; EDSS, Expanded Disability Status Scale; FSMC, Fatigue Scale for Motor and Cognitive Functions; n, number*.

**Table 3:** Data of study group separated according to the presence of cognitive fatigue.

	No cognitive fatigue (FSMC cognition ≤22)	Cognitive fatigue (FSMC cognition ≥22)
Number (*n*)	20	75
Gender (male:female)	8:12	28:47
Age^a^	48.15 ± 10.74	49.46 ± 9.99
Disease duration (years)^a^	9.56 ± 8.01	13.56 ± 9.95
EDSS score^a^	3.08 ± 2.30	4.03 ± 2.28
Cognitive fatigue subscore of the FSMC^a^	16.85 ± 2.90	35.36 ± 7.20
BDI total score^a^	2.75 ± 2.30	6.39 ± 3.41
COMPASS-31 score^a^	12.70 ± 12.08	23.63 ± 10.25
Orthostatic intolerance subscore COMPASS-31^a^	2.50 ± 3.05	5.08 ± 2.91
Vasomotor subscore COMPASS-31^a^	0.70 ± 1.50	1.25 ± 1.59
Secretomotor subscore COMPASS-31^a^	1.05 ± 1.57	1.60 ± 1.69
Gastrointestinal subscore COMPASS-31^a^	4.10 ± 4.80	6.73 ± 5.10
Bladder subscore COMPASS-31^a^	1.40 ± 2.09	3.05 ± 2.44
Pupillomotor subscore COMPASS-31^a^	2.95 ± 3.59	5.91 ± 3.47

*^a^Mean ± SD*.

*BDI, Beck Depression Inventory; COMPASS-31, Composite Autonomic Symptom Scale-31; EDSS, Expanded Disability Status Scale; FSMC, Fatigue Scale for Motor and Cognitive Functions; n, number*.

Due to the heterogeneity of the patient group, we checked for differences between the rr MS, sp MS, and pp MS patients on the cognitive score of the FSMC, and the scales of the COMPASS-31 using one-way ANOVA and Kruskal–Wallis *H* test, respectively (see also Table 4). We found no significant difference between groups for the cognitive Fatigue Scale of the FSMC, the COMPASS-31 total score, and its subscores apart from the gastrointestinal subscore [χ^2^(2) = 7.86, *p* = 0.020] and the bladder subscore [χ^2^(2) = 11.85, *p* = 0.003]. Dunn–Bonferroni *post hoc* tests revealed that the rr MS and the sp MS patients differed significantly in the gastrointestinal subscore (rr MS: mean rank 44.83; sp MS mean rank: 60.91; *z* = −2.77, *p* = 0.005) and bladder subscore (rr MS: mean rank 39.06; sp MS mean rank: 61.07; *z* = −3.26, *p* = 0.003).

**Table 4:** Data of study group separated according to the disease course.

	rr MS	sp MS	pp MS
Number (*n*)	53	21	18
Gender (male:female)	20:33	11:10	4:14
Age^a^	45.71 ± 8.88	54.19 ± 9.55	54.67 ± 8.36
Disease duration (years)^a^	10.94 ± 8.73	19.95 ± 8.87	10.44 ± 9.78
EDSS score^a^	2.87 ± 1.96	5.67 ± 1.73	4.83 ± 2.11
Cognitive fatigue subscore of the FSMC^a^	31.79 ± 10.61	33.00 ± 8.73	29.72 ± 9.35
BDI total score^a^	10.80 ± 5.64	12.57 ± 6.28	11.75 ± 8.09
COMPASS-31 score^a^	19.83 ± 11.87	24.57 ± 11.47	21.95 ± 8.88
Orthostatic intolerance subscore COMPASS-31^a^	4.53 ± 3.15	4.48 ± 3.12	4.72 ± 3.08
Vasomotor subscore COMPASS-31^a^	0.98 ± 1.51	1.33 ± 1.80	1.22 ± 1.55
Secretomotor subscore COMPASS-31^a^	1.32 ± 1.73	2.19 ± 1.72	1.22 ± 1.34
Gastrointestinal subscore COMPASS-31^a^	5.30 ± 4.86	8.43 ± 4.77	6.06 ± 5.64
Bladder subscore COMPASS-31^a^	2.11 ± 2.30	3.91 ± 2.45	3.22 ± 2.39
Pupillomotor subscore COMPASS-31^a^	5.58 ± 3.76	4.23 ± 3.10	5.50 ± 3.79

*^a^Mean ± SD*.

*BDI, Beck Depression Inventory; COMPASS-31, Composite Autonomic Symptom Scale-31; EDSS, Expanded Disability Status Scale; FSMC, Fatigue Scale for Motor and Cognitive Functions; n, number*.

The results of the exploratory correlational analyses showed no correlation of the cognitive fatigue subscore with age, disease duration, or the level of disability. Also, the total score of the COMPASS-31 did not correlate with age, disease duration, or the level of disability. Concerning the domain subscores of the COMPASS-31, the bladder domain subscore significantly correlated with age (*r* = 0.38, *p* < 0.001), disease duration (*r* = 0.29, *p* = 0.003), and the level of disability (*r* = 0.47, *p* < 0.001). The gastrointestinal and the pupillomotor domain subscores significantly correlated with the EDSS score (*r* = 0.29, *p* = 0.002; *r* = −0.27, *p* = 0.005).

We found a strong positive correlation of the cognitive fatigue subscore with the total COMPASS-31 score (*r* = 0.47, *p* < 0.001; see Figure 1). Furthermore, the level of cognitive fatigue as well as the total COMPASS-31 score correlated with the mental BDI subscore (cognitive fatigue: *r* = 0.45, *p* < 0.001; Compass-31: *r* = 0.49, *p* < 0.001). When controlling for mental BDI score, the correlation of the cognitive fatigue subscore with the total COMPASS-31 score remained highly significant (*r* = 0.40; *p* < 0.001).

**Figure 2:**
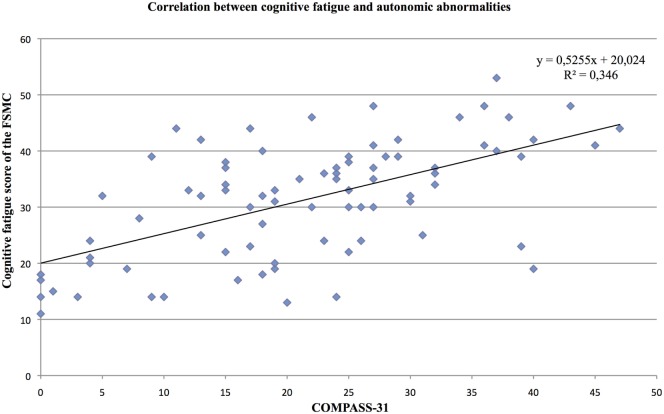
Correlation between the cognitive fatigue subscore of the Fatigue Scale for Cognitive and Motor Functions and the total score of the Composite Autonomic Symptom Scale-31, assessed in 95 MS patients.

**Figure 3:**
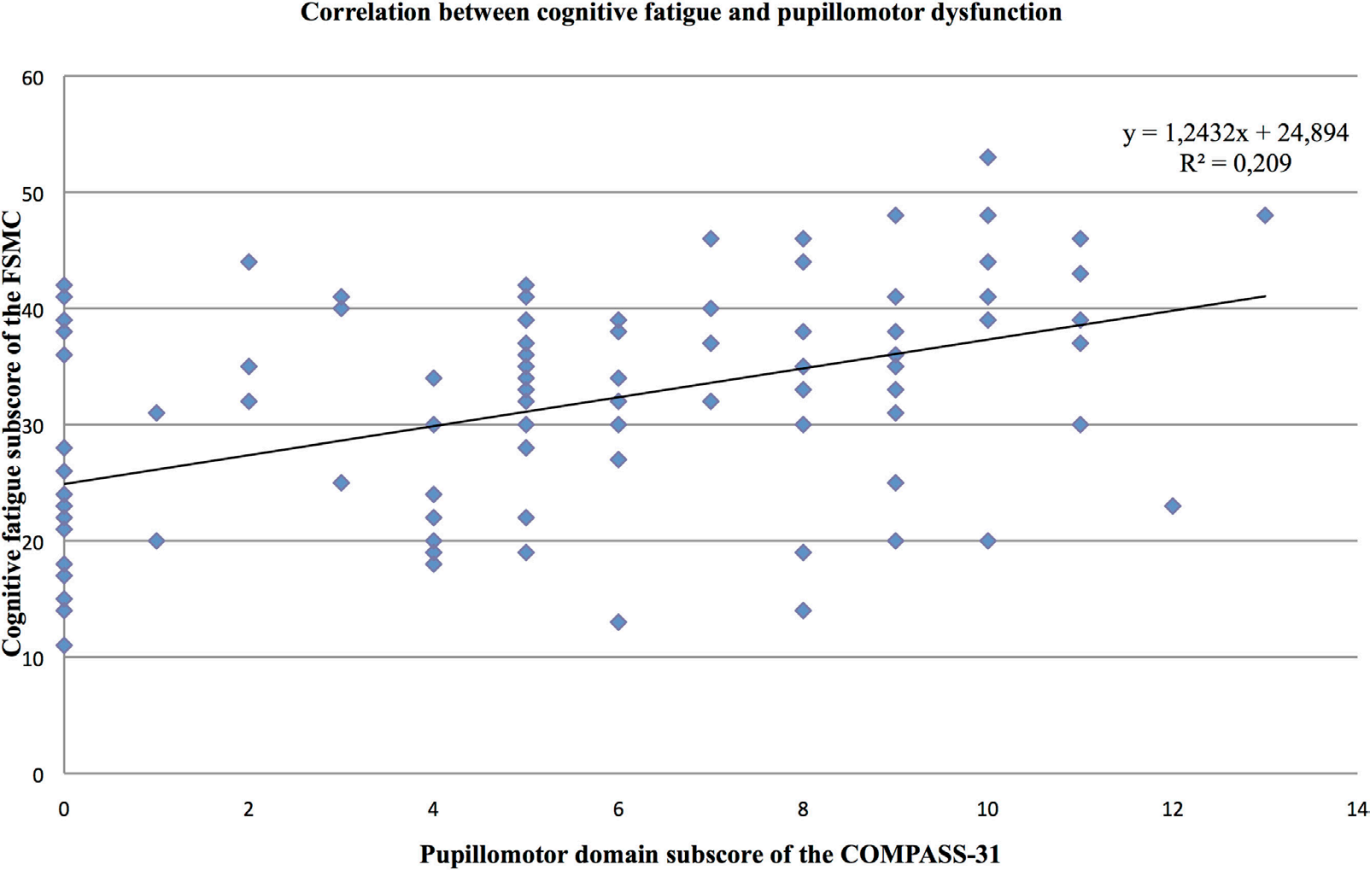
Correlation between the cognitive fatigue subscore of the Fatigue Scale for Cognitive and Motor Functions and the pupillomotor domain score of the Composite Autonomic Symptom Scale-31, assessed in 95 MS patients.

**Figure 4:**
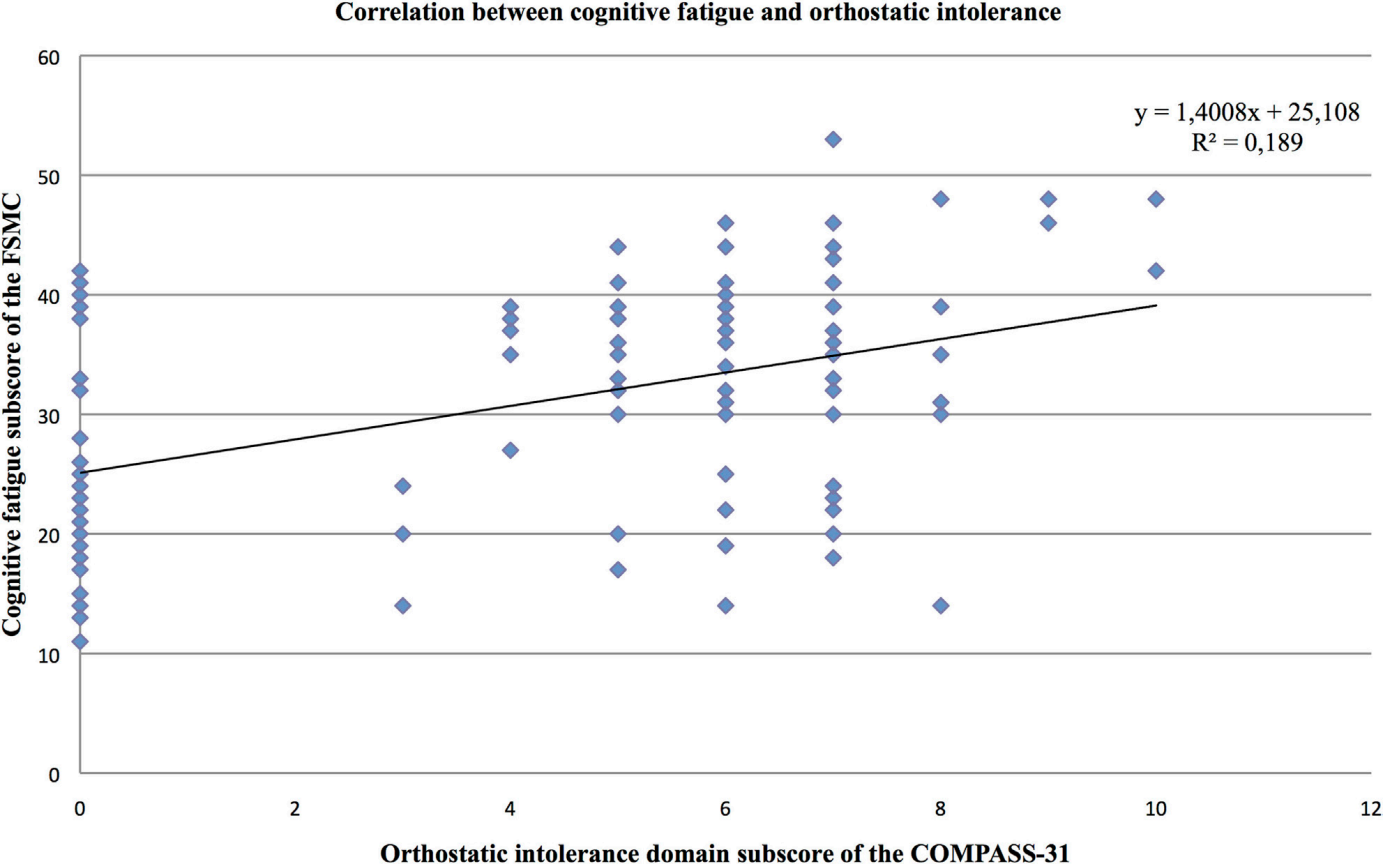
Correlation between the cognitive fatigue subscore of the Fatigue Scale for Cognitive and Motor Functions and the orthostatic domain score of the Composite Autonomic Symptom Scale-31, assessed in 95 MS patients.

**Figure 5:**
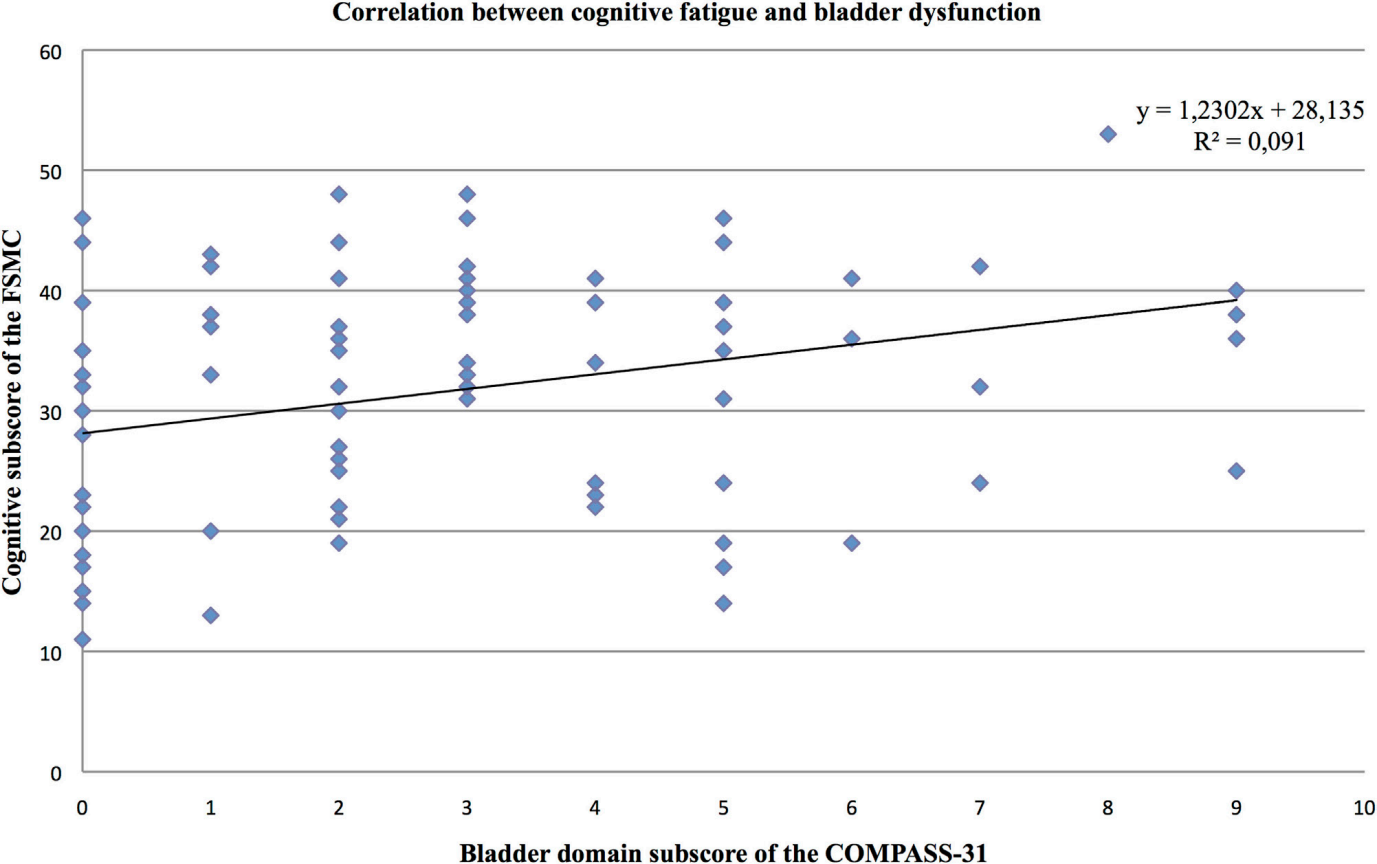
Correlation between the cognitive fatigue subscore of the Fatigue Scale for Cognitive and Motor Functions and the bladder domain score of the Composite Autonomic Symptom Scale-31, assessed in 95 MS patients.

Furthermore, the partial correlation analysis (controlling for mental BDI score) revealed positive correlations of the cognitive fatigue subscore with the pupillomotor (*r* = 0.39; *p* < 0.001), the orthostatic intolerance (*r* = 0.37; *p* < 0.001), and the bladder domain subscore (*r* = 0.23; *p* = 0.013; see Figures 2–5).

88 patients provided responses to all items considered in the regression analysis. The results of the forward regression analysis controlling for age, disease duration, EDSS, and mental BDI score revealed that the mental BDI score, the pupillomotor, the orthostatic intolerance, and the bladder domain of the COMPASS-31 were described as significant predictors for the cognitive fatigue score (see Table 5). The model describes a significant percentage of the variance of the level of cognitive fatigue (*R*^2^ = 0.47, *F* = 10.45, *p* < 0.001). This model describes much more of the variance (*R*^2^ = 0.47) than the model, including the control variables only (*R*^2^ = 0.26).

**Table 5:** Results of the model best predicting the cognitive fatigue score.

	Standardized coefficient beta	*t*	*p*
(Constant)		5.186	0.000
Age	−0.178	−1.830	0.071
Disease duration	0.120	1.339	0.184
EDSS	−0.020	−0.186	0.853
Mental BDI score	0.307	3.363	0.001
Pupillomotor domain score	0.242	2.399	0.019
Orthostatic domain score	0.273	2.997	0.004
Bladder domain score	0.201	2.060	0.043

*Dependent variable: cognitive fatigue score of the Fatigue Scale for Motor and Cognitive Functions*.

*Control variables: age, disease duration*.

*EDSS, Expanded Disability Status Scale; mental Beck Depression Inventory Scale (BDI) score*.

The regression analysis for the motor fatigue subscore as dependent variable showed that the mental BDI score (beta coefficient = 0.39, *p* < 0.001), the pupillomotor (beta coefficient = 0.24, *p* = 0.18), and orthostatic domain score (beta coefficient = 0.20, *p* = 0.04) of the COMPASS-31 best predicted the level of motor fatigue in MS patients (*R*^2^ = 0.41, *F* = 9.61, *p* < 0.001).

## Discussion

The results of this study point to a strong relationship between subjective cognitive fatigue (the experienced cognitive fatigue reported by questionnaires without objective measures) and autonomic abnormalities in MS patients. Especially, autonomic abnormalities, such as pupillomotor dysfunctions, orthostatic intolerance, and bladder dysfunctions, appear to be related to cognitive fatigue in MS patients. The strong relationship between cognitive fatigue and autonomic abnormalities might indicate that common pathological mechanisms are involved in cognitive fatigue and autonomic dysregulations in MS patients.

Concerning the relationship between fatigue and autonomic dysfunctions, particularly orthostatic intolerance seems to be related to fatigue in MS patients. Flachenecker and colleagues (3) examined heart rate variability of 60 MS patients during standard autonomic function tests and found that autonomic responses correlated with fatigue resembling a hypoadrenergic orthostatic response. Lebre et al. (4) investigated 50 rr MS patients and found that especially patients with fatigue present a loss in the capacity to increase blood pressure. Kanjwal and colleagues (14) investigated nine MS patients with postural orthostatic tachycardia syndrome and found that this syndrome was strongly related to fatigue and that treatment of postural orthostatic tachycardia resulted in a reduction of fatigue in the majority of investigated patients. Similar to our results, Cortez and colleagues (1) found a correlation between self-reported fatigue and autonomic symptoms in MS patients. Their results also pointed to a particular relation between fatigue and orthostatic intolerance in MS patients.

Bladder dysfunctions are the most common autonomic disturbances in MS (15). Most common urodynamic symptoms are nocturia, urinary urgency, and frequency (16). Urinary symptoms are correlated with disability status and considered to be related to spinal cord lesions or lesions in cortical regions that control urinary tract regulation (medial prefrontal cortex, insula, and pons) (17). Nevertheless, urinary symptoms, such as detrusor overactivity, detrusor-sphincter dyssynergia are already present in early MS stages and have been observed in several patients at the level of clinically isolated syndrome (CIS) (18). This might indicate that also other mechanisms than demyelination and atrophy contribute to these symptoms, especially in these early stages.

The same holds for pupillomotor dysfunctions. Pupillary disturbances such as abnormal pupillary light reflex latencies and abnormal contraction amplitudes have been frequently observed in early stages of MS (19–21), pointing to other mechanisms than increased demyelination and brain atrophy in the generation of these disturbances.

We found that autonomic symptoms, such as pupillomotor dysfunction, orthostatic intolerance, and bladder dysfunction, best predict the level of cognitive fatigue in MS patients. Since we included the variables disease duration and EDSS in the regression analysis, the results indicate that autonomic dysfunctions have a negative influence on the manifestation of cognitive fatigue independent of disease duration and progression. Furthermore, we included the mental BDI score in the regression analysis. Hence, it seems unlikely that the obtained results arise from a simple “over-reporting” of symptoms.

Overall, fatigue and autonomic abnormalities have been frequently described early in the disease course of MS (18, 20, 22). Already at the stage of a CIS and in very early MS stages, patients may present fatigue, bladder dysfunctions, and pupillary abnormalities (18, 20, 21, 23). During these early stages, neurological impairment is usually very limited and hardly any structural changes in the central nervous system have occurred yet. Consequently, factors such as increased brain atrophy, advanced disability, or increased age can be excluded as possible causes for fatigue and autonomic abnormalities in such early stages. However, very early stages of MS seem to reflect the same full-blown pattern of immunological abnormalities that is seen in later disease stages, highlighting the potential role of increased proinflammatory cytokines in the generation of subjective fatigue and autonomic abnormalities in MS patients (24).

Hanken et al. (6) developed a model of MS-related fatigue, based on all sorts of data. According to this model, the increased level of peripheral proinflammatory cytokines activates afferents of the vagus nerve that convey information about bodily inflammation to the central nervous system. This increased inflammation-induced activity of the afferent vagus nerve results in an increased activity in interoceptive brain areas and subsequently increases activity of the efferent vagus nerve. The increased activity in interoceptive brain areas might generate the feeling of fatigue, whereas the increased activity of the efferent vagus nerve that mainly contribute to the parasympathetic nervous system might lead to disruptions in autonomic functioning, whereas particularly those functions should be affected that are under direct control of the efferent vagus nerve.

Parasympathetic overactivity and resulting impaired sympathetic-mediated control might cause a reduction in blood pressure resulting in orthostatic intolerance, an increased promotion in voiding resulting in bladder dysfunction, and a constriction of the pupil resulting in problems with focusing.

The fact that the correlation between cognitive fatigue, on the one hand, and secretomotor and vasomotor symptoms, on the other hand, was not significant might be due to the fact that these functions are not completely under the control of the efferent vagus nerve. Sudomotor and vasomotor processes, for example, are only controlled by the sympathetic branch (25). Therefore, it is interesting that these two symptoms did not remain in the regression analysis, whereas the three autonomic function failures, which contributed to the prediction of fatigue level, are all influenced by the parasympathetic branch of the autonomic nervous system. This finding fits well with our model. The gastrointestinal domain stands under a complex homeostatic regulation, with inhibitory as well as excitatory parasympathetic nervous control, which might explain the lack of a correlation between this domain and the cognitive fatigue score (25). With respect to the bladder and gastrointestinal functions, the only autonomic function failures that differed between rr MS and sp MS, it should be noted that spinal lesions, among other factors, might also impair autonomic functions and that axons for bladder (and gastrointestinal) control travel through almost the entire spinal cord. Therefore, the chance of suffering spinal lesions during the disease development is much higher for these functions than for other autonomic functions. However, a more detailed mapping of specific autonomic nervous dysfunctions on cognitive fatigue would be highly speculative and cannot be answered by our data.

Assuming that systemic inflammation causes fatigue and autonomic dysfunctioning in MS patients, anti-inflammatory approaches such as IL-1β antagonists might reduce vagus nerve signaling and may, consequently, result in a reduction of fatigue and autonomic abnormalities (9). Consequently, IL-1β antagonists point to a completely new path for the treatment of fatigue. However, this assumption is rather speculative and more studies investigating the relation between fatigue, autonomic dysfunctions, and inflammation are needed to clarify this possibility.

A limitation of this study is that only questionnaires were used to investigate fatigue and autonomic functions. Objective measures of autonomic functions and cognitive fatigue are necessary to analyze the exact relationship between these symptoms. To check for the involvement of proinflammatory cytokines in the generation of these symptoms, blood investigations combined with fatigue and autonomic function measures need to be performed. Moreover, the COMPASS-31 does not cover the assessment of the complete range of autonomic symptoms. Abnormalities in sexual functions or thermoregulatory functions are not addressed by the questionnaire. MS patients often report sexual dysfunctions, such as sexual arousal disorders in women, erectile dysfunction in men, orgasmic dysfunction and dyspareunia. These dysfunctions also seem to be associated with MS-related fatigue (26). In addition, studies demonstrated an elevated body temperature in fatigued MS patients, pointing to thermoregulatory abnormalities in fatigued MS patients (27, 28). Hence, abnormalities within these autonomic functions should also be addressed to get a complete overview of the relation between autonomic functions and fatigue in MS patients.

It should be mentioned that this is not the only possible explanation for the occurrence of cognitive fatigue in MS. There are many causes besides autonomic functions but even when controlling for age, disease duration, and EDSS relations to autonomic functions remained in the model. Discussing all possible causes would be highly speculative and, therefore, we focus on the above named model.

Furthermore, it should be noted that our patient group was a mixed cohort, including patients suffering from rr MS, sp MS, and pp MS. As there might be different underlying pathological mechanisms for these three types, we still think that it is a representative patient group because in all of them inflammation plays a more or less central role (29).

In conclusion, we found a strong correlation between subjective feelings of cognitive fatigue and autonomic abnormalities in MS patients. Particularly, pupillomotor abnormalities, orthostatic intolerance, and bladder dysfunctions were correlated with cognitive fatigue, pointing to a common pathological mechanism in the generation of autonomic abnormalities and cognitive fatigue in MS.

## Ethics Statement

This study was carried out in accordance with the recommendations of the ethical board of the Medical Society in Bremen with written informed consent from all subjects. All subjects gave written informed consent in accordance with the Declaration of Helsinki. The protocol was approved by the ethical board of the Medical Society in Bremen.

## Author Contributions

CS: executing the study, assessment of study participants, and help on manuscript writing. HH: help on planning the study, help on statistical analysis, and help on manuscript writing. H-PS: help on planning the study. PE: help on writing the manuscript. KH: planning the study, statistical analysis, and manuscript writing.

## Conflict of Interest Statement

The authors confirm that there are no conflicts of interest, no sponsoring and patent holdings related to this article. The reviewer, ES, and handling editor declared their shared affiliation.

## Abbreviations

BDI, Beck’s Depression Inventory; CIS, clinically isolated syndrome; COMPASS-31, Composite Autonomic Symptom Scale-31; EDSS, Expanded Disability Status Scale; FSMC, Fatigue Scale for Motor and Cognitive Functions.
